# The Men’s Training Cup Keep Training: a masturbation aid improves intravaginal ejaculatory latency time and Erection Hardness Score in patients who are unable to delay ejaculation

**DOI:** 10.1093/sexmed/qfac010

**Published:** 2023-01-18

**Authors:** Masato Shirai, Keisuke Ishikawa, Ippei Hiramatsu, Kazuhiko Mizushima, Takamitsu Tsuru, Makoto Kurosawa, Akimasa Kure, Yuka Uesaka, Taiji Nozaki, Akira Tsujimura

**Affiliations:** Department of Urology, Juntendo University Urayasu Hospital, 2-1-1 Tomioka, Urayasu, Chiba 279-0021, Japan; Department of Urology, Juntendo University Urayasu Hospital, 2-1-1 Tomioka, Urayasu, Chiba 279-0021, Japan; Department of Urology, Graduate School of Medicine, Juntendo University, 2-1-1 Hongo, Bunkyo-ku, Tokyo 113-8431, Japan; Department of Urology, Juntendo University Urayasu Hospital, 2-1-1 Tomioka, Urayasu, Chiba 279-0021, Japan; Department of Urology, Graduate School of Medicine, Juntendo University, 2-1-1 Hongo, Bunkyo-ku, Tokyo 113-8431, Japan; Department of Urology, Juntendo University Urayasu Hospital, 2-1-1 Tomioka, Urayasu, Chiba 279-0021, Japan; Department of Urology, Juntendo University Urayasu Hospital, 2-1-1 Tomioka, Urayasu, Chiba 279-0021, Japan; Department of Urology, Graduate School of Medicine, Juntendo University, 2-1-1 Hongo, Bunkyo-ku, Tokyo 113-8431, Japan; Department of Urology, Juntendo University Urayasu Hospital, 2-1-1 Tomioka, Urayasu, Chiba 279-0021, Japan; Department of Urology, Juntendo University Urayasu Hospital, 2-1-1 Tomioka, Urayasu, Chiba 279-0021, Japan; Department of Urology, Juntendo University Urayasu Hospital, 2-1-1 Tomioka, Urayasu, Chiba 279-0021, Japan; Department of Urology, Juntendo University Urayasu Hospital, 2-1-1 Tomioka, Urayasu, Chiba 279-0021, Japan; Department of Urology, Juntendo University Urayasu Hospital, 2-1-1 Tomioka, Urayasu, Chiba 279-0021, Japan

**Keywords:** premature ejaculation, masturbation, masturbation aid device, Men’s Training Cup Keep Training, erection hardness

## Abstract

**Introduction:**

Premature ejaculation (PE) has negative personal consequences, such as distress, bother, frustration, and/or the avoidance of sexual intimacy. In Japan, no oral drugs or devices are approved or used clinically to treat PE. The Men’s Training Cup Keep Training (MTCK), a masturbation aid, was developed for PE. MTCK offers 5 grades of tightness and strength.

**Aim:**

We aimed to investigate the efficacy of the MTCK in patients who are unable to delay ejaculation.

**Methods:**

Inclusion criteria were 20- to 60-year-old men feeling distressed and frustrated by PE and who had the same sexual partners throughout the study period. Exclusion criteria were neurologic disease and uncontrolled diabetes mellitus, as well as the use of antidepressants, α-blockers, and 5α-reductase inhibitors. The protocol comprised an 8-week training period with the MTCK from level 1 to level 5, with each level used twice before moving to the next level.

**Outcome Measures:**

The main outcome measure was the extension of intravaginal ejaculation latency time (IELT). The secondary outcome measures were score improvements on the Premature Ejaculation Diagnostic Tool, Sexual Health Inventory for Men, Erection Hardness Score, and Difficulty in Performing Sexual Intercourse Questionnaire–5.

**Results:**

We enrolled 37 patients, and after 19 patients withdrew, 18 concluded the study without experiencing any adverse events. The mean patient age was 39.9 years. Geometric IELT after the 8-week training with the MTCK increased significantly (mean ± SE; 232.10 ± 72.16 seconds) vs baseline (103.91 ± 50.61 seconds, *P* = .006). Mean scores on the Premature Ejaculation Diagnostic Tool, Difficulty in Performing Sexual Intercourse Questionnaire–5, and Erection Hardness Score after 8-week training improved significantly vs the baseline values. The mean score on the Sexual Health Inventory for Men did not improve significantly after the 8-week training, but domain 1 did significantly improve after 8 weeks of MTCK use.

**Clinical Implications:**

The MTCK may be one possible treatment option for patients who are unable to delay ejaculation.

**Strengths and Limitations:**

This is the first study to show that the MTCK is effective for patients who are unable to delay ejaculation. A major limitation is that the present study was not strictly limited to an IELT <3 minutes.

**Conclusions:**

The MTCK may offer benefits not only for delay of ejaculation but also for erectile function.

## Introduction

Epidemiologic data have revealed that the prevalence of premature ejaculation (PE) is approximately 5% in the general population.^1^ PE has negative personal consequences, such as distress, bother, frustration, and/or the avoidance of sexual intimacy.^1^ An American Urological Association guideline for PE recommend selective serotonin reuptake inhibitors and topical anesthetics as the first-line agents of choice in the treatment of PE.^2^ However, pharmacotherapy has negative impacts, such as being inaccessible and expensive and causing side effects, as well as its inability to cure PE and its high cessation rate.^3^

Sexual and behavioral therapy is an alternative strategy for the treatment of PE with advantages that include no side effects, patients and partners learning sexual skills, intrapsychic examination, and the addressing of interpersonal and cognitive issues.^4^ Behavioral therapy involves becoming aware of pelvic floor muscles and learning how to time the execution of and maintain pelvic floor muscle contractions when sensing the preorgasmic phase. Repeated sessions of behavioral therapy are also used to prove additional pelvic floor rehabilitation.^5^^,^^6^ One previous study revealed that mean intravaginal ejaculation latency time (IELT) increased from 39.8 to 146.2 seconds after 12-week behavioral therapy. At the 6-month follow-up, the patients had maintained a significantly increased IELT of 112.6 seconds.^5^ Another study revealed that among patients completing behavioral therapy, 54% were cured of PE, and over time they learned how to postpone their ejaculation reflex.^6^

The main barriers to sexual and behavioral therapy for PE are accessibility, counseling fees, and weak evidence of the benefits of therapy.^3^ Ideal therapy for PE would include curability, accessibility, few side effects, low cost, and the ability for patients with PE to train themselves. The combination of a masturbation device with behavioral therapy is a new line of treatment for PE. In a randomized controlled clinical trial comprising 2 parallel groups, cognitive-behavioral treatment (CBT) alone vs CBT in conjunction with the Flip Zero masturbation device (Tenga Healthcare, Inc) revealed that geometric IELT was significantly improved in both groups when compared with baseline times. Moreover, when compared with CBT alone, CBT with the device significantly improved IELT.^7^ This study revealed that the Premature Ejaculation Profile was significantly improved by CBT with a device.^7^ Because the Flip Zero is reusable, it requires maintenance, such as washing and drying immediately after ejaculation. Furthermore, its tightness and strength can be variably adjusted such that the patient can control tightness that is too strong or strength that is too weak.

**Figure 1:**
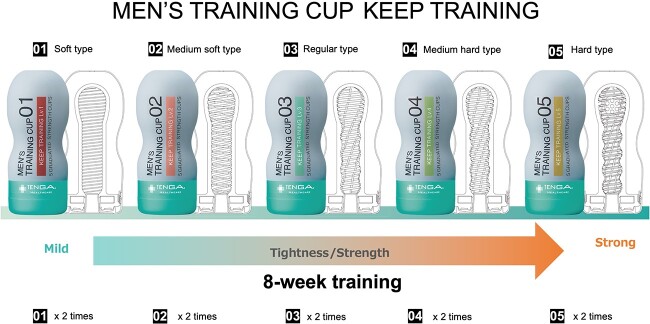
The 5 levels of tightness and strength of the Men’s Training Cup Keep Training (MTCK). Level 1 is the softest type with mild tightness, and level 5 is the hardest type with the strongest tightness. The protocol comprises an 8-week training period in which the patient uses the MTCK from level 1 to level 5, with the device used twice at each level before moving to the next level. The photo is reprinted with permission of TENGA Healthcare Inc.

The masturbation aid Men’s Training Cup Keep Training (MTCK; Tenga Healthcare, Inc) was developed specifically for PE. The MTCK offers 5 grades of tightness and strength. Level 1 offers the softest strength with mild tightness, and level 5 provides the hardest strength and strongest tightness (Figure 1). The MTCK is marketed as the Men’s Training Cup Endurance Training in the global market. Because the MTCK offers multiple steps and is disposable, it is easy for the patient to see his progress in treatment, it is more hygienic, and the patient enjoys differing levels of tightness. The inside pressure of the MTCK can also be regulated by covering a hole at the top of the device with a finger.

**Table 1:** Patient demographics at baseline (N = 18).

	**Mean ± SD or No.**
Age, y	39.87 ± 12.10
Weight, kg	66.71 ± 7.82
Height, cm	171.88 ± 5.25
Body mass index, kg/m^2^	22.55 ± 2.14
Smoking history	2
History	1: AGA + ED 1: LUTS 1: Hypertension
Medications	1: Sil + Tad + Fin 1: Tad 1: Nif

Abbreviations: AGA, androgenetic alopecia; ED, erectile dysfunction; Fin, finasteride; LUTS, lower urinary tract symptoms; Nif, nifedipine; Sil, sildenafil; Tad, tadalafil.

In Japan, because no oral drugs or devices are approved and used clinically to treat PE, we conducted clinical research into PE using the MTCK. We aimed to determine the following: does the MTCK increase ejaculation latency time? Therefore, we investigated the efficacy of the MTCK in improving ejaculations in patients who are unable to delay ejaculation.

## Methods

The main outcome measure was the extension of IELT as measured by a stopwatch. The secondary outcome measures were score improvements on the Premature Ejaculation Diagnostic Tool (PEDT), Sexual Health Inventory for Men (SHIM), Erection Hardness Score (EHS), and Difficulty in Performing Sexual Intercourse Questionnaire–5 (DPSIQ-5). All patients were examined by a medical doctor for evaluation of their conditions and adverse events. The anticipated risk of the aid device is irritation of penile skin.

### Measurement

The IELT measures the time from the beginning of vaginal intromission to that of intravaginal ejaculation. Stopwatch-measured IELT is frequently used as a means of quantifying the clinical response to therapy.^8^ We also measured the fold increase in IELT, which was calculated by dividing the geometric mean of the 8-week IELTs by the geometric mean of the baseline IELTs.

The PEDT is 5-item self-questionnaire that assesses ejaculation control, frequency of ejaculation, minimal necessary sexual stimulation, distress caused by PE, and interpersonal difficulty resulting from PE. The first 3 items cover the concept of control, but items 2 and 3 also assess frequency and minimal sexual stimulation, respectively. Items 4 and 5 cover distress and interpersonal difficulty caused by PE.^9^

In clinical practice and research, the SHIM is widely used to screen and diagnosis erectile dysfunction (ED) and its severity. The positive impact on understanding and improving male sexual function has been confirmed by the quantity and quality of research already published on the SHIM.^10^

The EHS examines a single-item patient-reported outcome for scoring erection hardness. A previous study revealed the EHS to be a simple, reliable, and valid tool for assessing erection hardness in clinical trials research.^11^

The DPSIQ-5 assesses the level of difficulty in performing sexual intercourse experienced by men with ED. Psychometric testing confirmed the use of the DPSIQ-5 as a complementary tool to existing patient-reported outcome tests used in clinical trials of ED.^12^

### Inclusion and exclusion criteria

Inclusion criteria were males aged 20 to 60 years who felt distressed and frustrated by PE and who had the same sexual partners throughout the study period. Excluded were patients who had uncontrolled diabetes mellitus or neurologic disease or took antidepressants, α-blockers, and 5α-reductase inhibitors.

### Ethical approval and informed consent

The present study protocol complied with good clinical practices and the Declaration of Helsinki (1996) and was in accordance with applicable institutional review board regulations. We registered the protocol in the University Hospital Medical Information Network–Clinical Trials Registry (UMIN000045057). The present study protocol, including all questionnaires, was reviewed and approved by the institutional review board of Juntendo University Urayasu Hospital (U20-0004). The study participants provided informed consent before the initiation of any study-related procedures and medications.

### Study protocol

The protocol comprised an 8-week training period with the MTCK from level 1 to level 5, with each level used twice before moving to the next level. Masturbation with the MTCK was performed by the participants while they paid attention to the control of their pelvic muscles and external urethral and anal sphincters, which was termed *sphincter control training*^8^ and consisted of 4 procedures.

Procedure 1: masturbation with the MTCK, paying attention to the pelvic muscles and external urethral and anal sphincters (week 1).

Procedure 2: masturbation with the MTCK, which included 4 active stops per exercise for a maximum of 45 seconds, relaxing the external urethral and anal sphincters at each stop (weeks 2-4).

Procedure 3: masturbation with the MTCK without cessation of stimulation, with 4 moments of relaxation by alternately tensing and relaxing the external urethral and anal sphincters before ejaculation (weeks 5 and 6).

Procedure 4: masturbation with the MTCK without cessation of stimulation, with 4 moments of relaxation by alternately tensing and relaxing the external urethral and anal sphincters before ejaculation with intercourse movements (weeks 7 and 8).^7^^,^^13^

MTCK devices were provided at no cost to the participants.

### Statistical analysis

Data are shown as mean ± SE. We determined statistical significance with a paired *t*-test and considered *P* < .05 to indicate statistical significance. We used SPSS Statistics for Windows (Japanese version 28; IBM Japan) for all statistical analyses.

## Results

Originally, 37 patients were enrolled, and after 19 withdrew from the study, 18 with a mean age of 39.9 years continued without experiencing any adverse events (for demographic information, see Table 1).

The geometric mean IELT after the 8-week training with the MTCK increased significantly (232.10 ± 72.16 seconds) vs the baseline time (103.91 ± 50.61, *P* = .006) (Table 2). The IELT improved in all cases except 1. The IELT in that case was 60 seconds at baseline and the same 60 seconds after training. The geometric mean of the fold increase in the IELT was 2.23 ± 3.35. Five patients had a doubling of their IELTs, 10 had an IELT >180 seconds, and 8 had an IELT >300 seconds.

**Table 2:** Change in outcome measures after 8-week training with the Men’s Training Cup Keep Training.^a^

**Outcome**	**Baseline**	**8-wk training**	***P* value**
Intravaginal ejaculation latency time, s	103.91 ± 50.61	232.10 ± 72.16	.006
**Questionnaire**			
Premature Ejaculation Diagnostic Tool	12.28 ± 1.24	8.44 ± 1.29	.004
Difficulty in Performing Sexual Intercourse Questionnaire–5	16.61 ± 0.95	19.39 ± 0.75	.004
Erection Hardness Score	3.00 ± 0.18	3.39 ± 0.14	.004
Sexual Health Inventory for Men	16.39 ± 0.69	17.33 ± 0.99	.173

^a^Data are presented as mean ± SE.

The mean PEDT score after the 8-week training improved significantly (8.44 ± 1.29) vs baseline (12.28 ± 1.24, *P* = .004).

After the 8-week training, the mean scores of the DPSIQ-5 and EHS improved significantly as compared with those at baseline (19.39 ± 0.75 vs 16.61 ± 0.95, *P* = .004; 3.39 ± 0.14 vs 3.00 ± 0.18, *P* = .004, respectively).

The mean SHIM score did not significantly improve after the 8-week training session (17.33 ± 0.99) as compared with the baseline value (16.39 ± 0.69, *P* = .173). However, the domain 1 score of the SHIM did significantly improve after the 8-week training session (*P* = .028) (Table 3).

**Table 3:** Change in domain scores of 3 sexual health questionnaires.

**Score: domain**	**Baseline**	**8-wk training**	***P* value**
Premature Ejaculation Diagnostic Tool			
1	2.44 ± 0.23	1.50 ± 0.20	<.001
2	2.44 ± 0.34	1.67 ± 0.35	.009
3	2.17 ± 0.35	1.72 ± 0.32	.134
4	2.61 ± 0.31	1.78 ± 0.33	.007
5	2.61 ± 0.24	1.78 ± 0.26	.009
Difficulty in Performing Sexual Intercourse Questionnaire–5			
1	4.06 ± 0.24	4.50 ± 0.19	.042
2	3.83 ± 0.22	4.17 ± 0.20	.138
3	3.22 ± 0.29	3.78 ± 0.22	.046
4	2.78 ± 0.26	3.56 ± 0.22	<.001
5	2.72 ± 0.25	3.39 ± 0.27	.006
Sexual Health Inventory for Men			
1	2.67 ± 0.21	3.11 ± 0.28	.028
2	3.78 ± 0.21	4.06 ± 0.19	.172
3	3.56 ± 0.23	3.78 ± 0.21	.298
4	3.06 ± 0.29	3.06 ± 0.37	>.99
5	3.33 ± 0.30	3.56 ± 0.26	.36

^a^Data are presented as mean ± SE.

We did not collect follow-up data on these patients after they completed the study.

## Discussion

We investigated the ability of training with the MTCK to improve ejaculation in patients who are unable to delay ejaculation. This is the first study, to our knowledge, to show that the MTCK is effective for patients who are unable to delay ejaculation. The data showed that the MTCK could clearly aid in reducing ejaculatory symptoms in the study patients. The IELT significantly improved with the MTCK, as did scores on the PEDT, EHS, and DPSIQ-5 (Table 2). Also, many domains of the questionnaires were significantly improved with MTCK use—including SHIM domain 1; PEDT domains 1, 2, 4, and 5; and DPSIQ-5 domains 1, 3, 4, and 5—indicating improvement in ejaculatory control, distress, interpersonal difficulty, erection hardness, maintenance of erection, and confidence of erection (Table 3). We speculated a possible effect for PE with the MTCK in that desensitization resulted due to the greater friction and pressure applied to the penis during exercises with the device. Furthermore, the device generated stimuli during training that are very similar to those produced in the penis during intercourse. A previous study revealed that ejaculation training with a masturbation aid stimulates the penile glans similar to that occurring during intercourse, which favors the transfer of the experience gained during masturbation to actual intercourse.^7^^,^^13^ It will be more beneficial to use the MTCK than not to use it because the MTCK masturbation device provides a condition similar to that of coitus; therefore, it may allow the participants to more easily transfer what they learned to coital relationships.^7^^,^^13^

The present study provided results comparable with those obtained with dapoxetine, clomipramine, topical anesthetics, tramadol, and phosphodiesterase type 5 inhibitor (PDE5i).^14^^,^^15^ Dapoxetine is short-acting selective serotonin reuptake inhibitor that provides on-demand treatment for PE.^14^ Dapoxetine use resulted in a significantly improved mean stopwatch-measured IELT of 216 seconds from baseline and placebo times of 54 and 114 seconds, respectively. Also, when compared with the placebo, dapoxetine significantly decreased the patients’ levels of personal distress related to ejaculation. The adverse events most commonly experienced were nausea (17.3%), dizziness (9.4%), and headache (7.9%).^14^

Clomipramine, a tricyclic antidepressant, resulted in an efficacious 4- to 6-fold increase in IELT and safety with on-demand or daily use. It significantly improved the mean PEDT score to 10.5 from baseline and placebo scores of 15.1 and 14.4, respectively. The most common adverse events similarly included nausea (15.7%) and dizziness (4.9%).^16^

Topical anesthetics such as lidocaine, prilocaine, and benzocaine are efficacious for PE. Mixed lidocaine-prilocaine sprays significantly extended the mean IELT to 156 seconds from a baseline time of 34 seconds and a placebo time of 48 seconds. Vulvovaginal burning in partners (7.8%), ED (5.4%), and penile hypoesthesia (4.9%) were the most common adverse events.^17^

PDE5i was revealed to have a role in the reduction of performance anxiety and fear of losing an erection. The combination therapy of paroxetine and tadalafil significantly improved the mean patient-estimated IELT to 175.2 seconds from the baseline time of 71.6 seconds and the paroxetine-alone time of 117.3 seconds.^15^ On the Index of Premature Ejaculation, PDE5i showed improvement in ejaculatory confidence, ejaculatory control, and sexual satisfaction when compared with placebo. Headache (15%), flushing (15%), and dyspepsia (5%) were the main adverse effects of PDE5i in PE management.^18^

Tramadol is an opiate analgesic with the dual actions of activating opioid receptors and inhibiting serotonergic and noradrenergic reuptake.^19^ Tramadol significantly improved the mean stopwatch-measured IELT to 351 seconds when compared with the placebo time of 81 seconds. Tramadol’s adverse events include the potential for abuse or addiction, constipation, nausea, headache, somnolence, and serotonin syndrome.^19^

We observed no side effects in the present study, which offers great benefit vs pharmacotherapy. For example, one of the main causes of discontinuing the dapoxetine pharmacotherapy for the treatment of PE was the drug’s side effects.^20^

The MTCK may offer benefits not only for delay of ejaculation but also for erectile function. We speculated that the structure of the MTCK offers benefit for ejaculation and penile erection. Patients can regulate the inside pressure of the MTCK. Especially, patients can create negative pressure inside the device similar to that of a vacuum erection device (VED). The VED is a leading modality frequently used in penile rehabilitation^21^ whose mode of operation is to increase blood flow into the corpus cavernosa. Antiapoptotic and antifibrotic processes are activated by VED therapy, which potentially preserves veno-occlusive mechanisms.^21^

PE and ED compete in a vicious cycle in which patient control of ejaculation instinctively reduces the excitation level, which can lead to ED, and the attempt to achieve an erection, which basically increases excitation and can lead to PE.^22^ We speculated that improvement of erection with the MTCK would have a positive effect on ejaculation similar to that of PDE5i.^15^ The MTCK would be part of a virtuous circle of erection and ejaculation.

Participants in the present study each had the same sexual partner for the duration of the study. However, it is not necessary for the partner to attend treatment for PE with the MTCK. Individual treatment of PE for patients without partners will be also developed. Furthermore, the MTCK can be used in an internet-based or online therapy program for PE.^23^

The present study has some limitations. First, this study has no control group (ie, behavioral therapy alone). We could not evaluate the behavioral effect on ejaculation without the MTCK. A previous study showed that a masturbation device combined with behavioral therapy significantly improved IELT and the Premature Ejaculation Profile when compared with behavioral therapy alone.^7^ After the Clinical Trials Act was enacted in Japan, clinical research, especially the performance of randomized controlled studies, has become very difficult. We will need to implement international collaboration in randomized clinical research for PE treatment with the MTCK.

Second, the sample size was small, and 19 of the 37 patients dropped out of the study. We could not determine a definite reason for discontinuation because these participants did not appear for reexamination. A previous study revealed that the main reasons for discontinuing PE treatments were cost (30%) and the need for a drug for each incidence of sexual intercourse (25%), but poor efficacy rated only 10%.^20^ We speculated that the participants who dropped out did so for reasons unrelated to cost or the need to use the MTCK at each incidence of sexual intercourse. Our treatment may not be broadly effective, as adherence was half at best.

Third, the study was not strictly limited to an IELT <3 minutes. The IELTs of 13 patients were <3 minutes at baseline. In all cases except 1, IELT following MTCK training was improved vs the baseline time. The IELT did not worsen when compared with the baseline time in any participant.

Fourth, the duration of the present study was 8 weeks, and follow-up of the treatment at 3 to 6 months was not performed. Thus, we could not evaluate long-term effects.

## Conclusions

This is the first study to show that the MTCK appears to be effective for patients who are unable to delay ejaculation. The MTCK may offer benefits not only for delay of ejaculation but also for erectile function, making it one possible treatment option for these patients.

## Data Availability

The data sets generated and analyzed during the current study are available in the figshare repository at https://doi.org/10.6084/m9.figshare.2013779.v1. *Conflicts of interest:* None declared.
